# Individual parkinsonian motor signs and striatal dopamine transporter deficiency: a study with [I-123]FP-CIT SPECT

**DOI:** 10.1007/s00415-019-09202-6

**Published:** 2019-01-28

**Authors:** Elina Mäkinen, Juho Joutsa, Elina Jaakkola, Tommi Noponen, Jarkko Johansson, Miia Pitkonen, Reeta Levo, Tuomas Mertsalmi, Filip Scheperjans, Valtteri Kaasinen

**Affiliations:** 1 Division of Clinical Neurosciences, University of Turku, Turku University Hospital, POB 52, 20521 Turku, Finland; 2 Department of Neurology, University of Turku, Turku University Hospital, Turku, Finland; 3 Athinoula A. Martinos Center for Biomedical Imaging, Massachusetts General Hospital, Harvard Medical School, Boston, MA USA; 4 Berenson-Allen Center for Noninvasive Brain Stimulation, Beth Israel Deaconess Medical Center, Harvard Medical School, Boston, MA USA; 5 Department of Nuclear Medicine, Turku University Hospital, Turku, Finland; 6 Department of Medical Physics, Turku University Hospital, Turku, Finland; 7 Turku PET Centre, University of Turku, Turku, Finland; 8 Department of Nuclear Medicine, Helsinki University Hospital, Helsinki, Finland; 9 Department of Neurology, Helsinki University Hospital, Helsinki, Finland; 10 Department of Clinical Neurosciences (Neurology), University of Helsinki, Helsinki, Finland

**Keywords:** Parkinsonism, Dopamine, [I-123]FP-CIT, SPECT, Rigidity, Facial expression

## Abstract

**Introduction:**

Total parkinsonian motor symptom severity correlates with presynaptic striatal dopamine function in patients with Parkinson’s disease. There is a lack of studies that have investigated the associations between parkinsonian motor signs and striatal dopaminergic deficiency in patients with parkinsonism of an unknown origin. Identification of specific motor signs associated with the highest likelihood of striatal dopamine deficiency could aid the differential diagnostics of parkinsonian and tremor syndromes.

**Methods:**

In this cross-sectional clinical and imaging study, detailed motor examinations were performed for 221 patients with parkinsonism or tremor of an unknown origin immediately before dopamine transporter (DAT) [I-123]FP-CIT SPECT imaging. Region-of-interest and voxel-based methods were used to investigate striatal DAT deficiency in relation to individual motor signs.

**Results:**

Upper extremity rigidity and facial expression were the only motor signs that differentiated patients with normal and abnormal striatal DAT function. The presence of any upper extremity rigidity showed the highest likelihood of DAT deficiency (OR 4.79, 95% CI 1.56–14.75, *P* = 0.006) followed by reduced facial expression (OR 2.14, 95% CI 1.14–4.00, *P* = 0.018). In patients with DAT deficits, reduced facial expression was associated with DAT deficiency specifically in the caudate nucleus, and increased upper extremity rigidity was associated with DAT loss in the dorsal putamen (FWE-corrected *P* < 0.05).

**Conclusions:**

Increased upper extremity muscle tone and hypomimia are independently associated with a higher likelihood of striatal hypodopaminergic imaging finding. This information can be used as a factor when the clinical need of auxiliary investigations, such as DAT SPECT, is considered for patients with parkinsonism.

**Electronic supplementary material:**

The online version of this article (10.1007/s00415-019-09202-6) contains supplementary material, which is available to authorized users.

## Introduction

There are numerous neurodegenerative and symptomatic causes of parkinsonism [1]. The many causal possibilities for parkinsonism are reflected in the suboptimal diagnostic accuracy of Parkinson’s disease (PD). A recent meta-analysis of clinicopathological studies demonstrated that the diagnostic accuracy of PD is approximately 75% among non-experts [2].

One possible method that could be used to improve diagnostic accuracy in parkinsonism of an unknown origin is presynaptic dopaminergic functional imaging. The uptake of putaminal presynaptic dopaminergic tracers in positron emission tomography (PET) and single photon emission computed tomography (SPECT) imaging is decreased by approximately 50% in patients with early to moderate PD, with practically no overlap when compared to healthy individuals [3]. In addition, the differentiation of patients with essential tremor (ET) from neurodegenerative parkinsonism with dopamine transporter (DAT) SPECT imaging has shown a high diagnostic accuracy [4]. [I-123]*N*-ω-fluoropropyl-2β-carbomethoxy-3β-(4-iodophenyl)nortropane ([I-123]FP-CIT) SPECT can also be used to establish the early diagnosis of neurodegenerative parkinsonism, in the differential diagnosis between dementia with Lewy bodies (DLB) and other dementias such as Alzheimer’s disease (AD), as well as between neurodegenerative parkinsonism with presynaptic dopamine loss (PD, DLB, multiple system atrophy MSA, progressive supranuclear palsy PSP and corticobasal syndrome CBS) and secondary parkinsonism, e.g., neuroleptic-induced parkinsonism [5]. However, its aid in the differentiation between vascular parkinsonism (VP) and PD remains partly contradictory [6]. Thus, dopaminergic imaging appears to represent a useful method for identifying neurodegenerative parkinsonism patients with striatal dopamine deficits, but the method is costly, has limited availability and is associated with radiation. Consequently, the clinical evaluation of parkinsonism patients remains the gold standard in most regions of the world.

The current evidence suggests an inverse correlation between parkinsonian motor symptom severity, particularly of bradykinesia and axial symptoms, and striatal DAT binding in patients with PD [7], whereas rigidity appears to correlate moderately and tremor only very weakly, or not at all, with striatal DAT binding [7, 8]. It should be noted, however, that a large proportion of the previous data has been collected in research studies that have investigated patients with established diagnoses of PD and healthy individuals, and thus, the results may convert poorly to a clinical setting when patients present with unclear or atypical parkinsonian motor symptoms. There is a lack of studies that have investigated the associations of individual parkinsonian motor signs with striatal dopamine deficiency in patients with clinical parkinsonism and tremor of an uncertain origin.

A widely used method in quantitative phenotyping of PD is the Unified Parkinson’s Disease Rating Scale (UPDRS), which revision, MDS-UPDRS was published in 2008 [9]. In the present study, we used functional DAT imaging and MDS-UPDRS motor scoring for 221 patients with parkinsonism or tremor of an unknown origin. The aims of the study were to identify parkinsonian motor signs that point to striatal dopaminergic deficiency, and to investigate whether these associations are particularly present in certain striatal subregions in patients with striatal DAT deficits.

## Patients and methods

### Patients and study description

This cross-sectional clinical and imaging study consisted of patients scanned with [I-123]FP-CIT SPECT because of parkinsonism or tremor of an unknown origin. The imaging was performed between the years of 2014–2017 at two centers. All patients were clinically examined 2–4 h before scanning. The examinations included a clinical interview, part III of the MDS-UPDRS [9], the original Hoehn and Yahr (H&Y) stage [10, 11] and the Mini-Mental State Examination (MMSE) [12]. Two-hundred and twenty-one consecutive patients willing to participate in the study and who were examined before February 2017 with complete MDS-UPDRS-III ratings and successful semi-quantitative scan analyses were included in the analyses. One-hundred and seventy-eight patients (80.5%) were scanned and examined in Center 1 and 43 (19.5%) in Center 2. Forty (18.1%) patients were receiving antiparkinsonian medications at the time of clinical examination and imaging. The study was approved by the Ethics Committees of the Hospital Districts, and was conducted according to the principles of the Declaration of Helsinki. Informed consent was obtained from all participants included in the study.

To investigate individual parkinsonian motor features, the MDS-UPDRS part III ratings were divided into six main subscores: bradykinesia, rigidity, tremor, axial signs, speech and facial expression, modified from the subscores from the original motor UPDRS ratings [7]. The toe tapping and freezing of gait items, which were not included in the original UPDRS [9], were included in the bradykinesia and axial signs subscores, respectively. Tremor was further categorized into rest, postural and kinetic tremors, and the asymmetry indices were calculated for the lateralized signs (Supplementary Table).

### SPECT imaging and image analyses

The SPECT imaging procedure and the image analyses, including the region-of-interest (ROI) analysis (BRASS, version 2.6H. by Hermes Medical solutions, Stockholm, Sweden) with scanner-specific corrections and patient age corrections for the specific binding ratio (SBR) calculations of six striatal ROIs (Supplementary Table 1), and the voxel-based image analysis (SPM12, http://www.fil.ion.ucl.ac.uk/spm/software/spm12/), are described in detail in the Supplementary Material.

The original interpretations of the scans by nuclear medicine physicians were reviewed, but the classification of patients into the normal and abnormal DAT binding groups was based on the automated semi-quantitative BRASS analysis. A scan was defined as abnormal if the semi-quantitative uptake was more than two standard deviations below the reference mean in any of the six analyzed regions [13]. Cases on the borderline of abnormality (*n* = 11) in the original evaluations were carefully re-evaluated, and a consensus decision was made by the investigators, based both on the visual interpretations and the initial and new semi-quantitative analyses with both age and camera corrections for the striatal SBRs [13], as described [14].

### Statistical analyses

The assumption of normality was evaluated visually from histograms together with Shapiro–Wilk tests. Mann–Whitney *U* tests and Chi-square tests were used to investigate group differences in the continuous and categorical variables, respectively. The Benjamini–Hochberg procedure was applied to control the probability of type I errors due to multiple comparisons of all the motor signs, with a false discovery rate of 5%. Binary logistic regression analyses were used to study whether the demographical and clinical factors that significantly differed between patients with and without striatal DAT deficiency had a higher likelihood of abnormal DAT binding. ORs for the motor signs were calculated for the presence of signs vs. no signs due to clinical interest. ROC curves for continuous upper extremity rigidity total score and facial expression score were plotted and areas under the curves (AUCs) were analyzed. Cut-off values were calculated for these variables, and for motor symptom duration from the corresponding ROC analyses by applying and choosing the maximum value of the Youden’s index [15]. One-way ANOVA was used to investigate differences in putamen and caudate DAT binding with respect to facial expression (0–4 points on the MDS-UPDRS) and mean upper extremity rigidity scores (0–0.5, 1–1.5, 2–2.5, 3–3.5, and 4 points), and to investigate differences in DAT binding between the SPECT scanners. Patients with the highest facial expression scores (3 and 4) and mean upper extremity rigidity scores (3–3.5 and 4) were combined because of the low number of patients with a score of 4. The equality of variances was tested with Levene’s test. Tukey HSD corrections were used to correct for multiple comparisons in the ANOVAs. *P* values less than 0.05 were considered significant. For all analyses, IBM SPSS Statistics Version 24 (IBM Corp., New York, USA) was used.

Finally, we investigated the striatal subregions where the motor signs showed associations with the imaging outcome (facial expression and upper extremity rigidity) stemmed from using the voxel-wise approach in SPM12. The analyses were restricted to the patients with abnormal DAT binding. One patient lacked the imaging data and was therefore left out of these analyses. The effect of hypomimia (scores of 2–4 vs. 0–1, *n* = 50 vs. *n* = 59) or mean upper extremity rigidity (scores of 2–4 vs. 0–1.5, *n* = 56 vs. *n* = 54) was investigated using univariate regression models and a multivariate regression model. A stringent statistical threshold was used, considering only voxel-level family wise error (FWE)-corrected *P* values less than 0.05 to be significant. The cluster coordinates are presented for all clusters with spatial extents of more than 5 voxels. All of the analyses were also conducted in the subsample of patients who were not receiving antiparkinsonian medications at the time of imaging and clinical examinations.

## Results

### Differences between patients with normal and abnormal striatal DAT binding

**Fig. 1:**
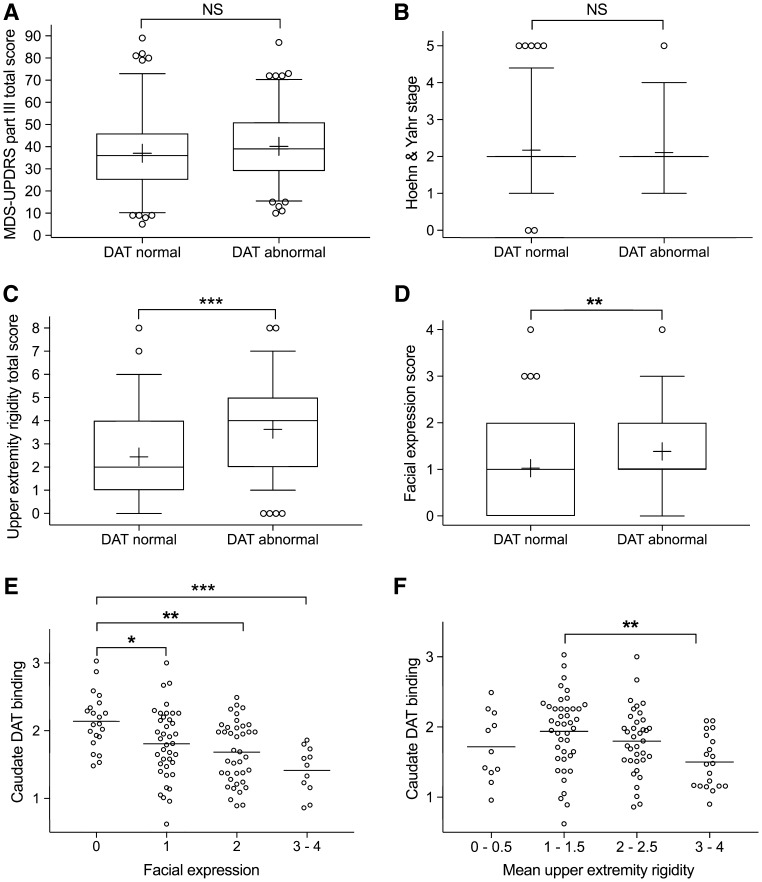
Group differences (**a**–**d**) and the effects of hypomimia and increasing upper extremity rigidity in patients with striatal DAT deficits (**e, f**). **a, b** There were no differences in the overall motor symptom severity between patients with normal and abnormal striatal DAT binding. **c, d** Upper extremity rigidity and facial expression were the only motor signs that differed between patients with and without striatal DAT deficits. Box plots and whiskers represent the medians and the 25–75 and 5–95 percentiles, respectively, and the means are shown as ‘+’. **e, f** In patients with striatal DAT deficits, reduced facial expressions were particularly associated with the reduced caudate nucleus DAT binding. **e***F* = 7.51, *P* < 0.001. 0 vs. 1, *P* = 0.029; 0 vs. 2, *P* = 0.001; 0 vs. 3–4, *P* < 0.001; 1 vs. 3–4, *P* = 0.068; 1 vs. 2, *P* = 0.64; 2 vs. 3, *P* = 0.33. **f***F* = 3.88, *P* = 0.011. 1–1.5 vs. 3–4, *P* = 0.006; 2-2.5 vs. 3–4, *P* = 0.12; rest pairwise post hoc comparisons, *P* > 0.43. Means are shown as lines. ***P < 0.001, **P < 0.01, *P < 0.05, *NS* non-significant

There were 110 patients with abnormal DAT binding and 111 patients with normal DAT binding. Patients with normal and abnormal DAT binding did not differ in terms of age, sex, education level, general cognitive capacity (MMSE), MDS-UPDRS total score (Fig. 1a) or H&Y stage (Fig. 1b) (Table 1). Patients with normal DAT binding had longer motor symptom durations than patients with abnormal binding (Table 1). When analyzing MDS-UPDRS-III subscores, speech, bradykinesia total score, axial signs, tremor total score (Table 1), and any tremor category (Supplementary Table 2) did not differ between patients with normal and abnormal DAT binding.

**Table 1:** Demographic and clinical characteristics, together with the motor examination results of 221 patients with parkinsonism or tremor of an unknown origin with and without striatal dopamine transporter (DAT) binding deficiency in [I-123]FP-CIT SPECT imaging

	DAT normal (*n* = 111)	DAT abnormal (*n* = 110)	*P* value
*Demographic and clinical characteristics*			
Age (years)	64.2 (12.2)	65.5 (9.4)	0.88
Sex (F/M)	54/57	55/55	0.84
Formal education (years)	12.6 (4.7)	13.2 (4.1)	0.19
MMSE score	26.2 (3.1)	26.6 (2.7)	0.27
Motor symptom duration at scan (years)	4.3 (5.8)	2.6 (3.7)	*0.013*
*Motor examination*			
Original Hoehn and Yahr stage	2.2 (0.9)	2.1 (0.9)	0.42
MDS-UPDRS part III total score	37.0 (17.4)	40.2 (15.9)	0.13
Facial expression	1.03 (0.89)	1.39 (0.92)	0.004*
Speech	0.81 (0.84)	0.96 (0.91)	0.22
Axial signs	4.19 (3.51)	4.46 (3.83)	0.70
Bradykinesia total score	16.60 (9.04)	18.10 (9.24)	0.27
Tremor total score	6.84 (5.28)	6.15 (4.39)	0.48
Rigidity total score	7.02 (4.53)	8.88 (4.62)	0.002*
Neck rigidity	1.44 (1.36)	1.75 (1.40)	0.09
Lower extremity rigidity total score	3.14 (2.36)	3.50 (2.42)	0.27
Upper extremity rigidity total score	2.44 (1.81)	3.63 (1.79)	< 0.001*

Mann–Whitney *U* tests were used to investigate continuous variables and Chi-square tests were used to investigate categorical variables. Values are presented as *n* or mean (SD) for demonstrative purposes. Numbers of missing values: education *n* = 3, MMSE *n* = 2, motor symptom duration *n* = 22, facial expression *n* = 1, axial symptoms *n* = 10, bradykinesia total score *n* = 3

*MMSE* Mini-Mental State Examination

*Significant after multiple comparisons correction using Benjamini–Hochberg procedure

Italic values indicate significance of *P* value (*P* < 0.05)

Patients with abnormal DAT binding had greater rigidity (total score) than patients with normal binding (Table 1). The difference in rigidity was significant in the upper extremities (48.8% higher mean upper extremity rigidity total scores in abnormal patients, Table 1; Fig. 1c) but not in the lower extremities or neck (Table 1). Patients with abnormal DAT binding scored higher also in terms of facial expression than patients with normal binding (Table 1; Fig. 1d). Asymmetry indices of motor signs, especially the asymmetry indices of rigidity and bradykinesia items, were higher in patients with abnormal DAT binding (Supplementary Table 2), but these results did not remain significant after the Benjamini–Hochberg procedure.

### Associations of rigidity and facial expression with DAT binding

The presence of any upper extremity rigidity (scores of 1–4 vs. 0 in at least one upper extremity) and the presence of any loss in facial expression (scores of 1–4 vs. 0 on the facial expression item) were associated with a higher likelihood of striatal DAT deficiency (Table 2). Each 1-year increase in the motor symptom duration was associated with a lower likelihood of DAT deficiency (Table 2). Upper extremity rigidity, hypomimia and motor symptom duration were all independently associated with abnormal or normal striatal DAT binding when included in the multivariate regression model (Table 2). The cut-off values were 2.5 points for upper extremity rigidity total score, 1.5 for facial expression score, and 1.58 years for motor symptom duration, and the AUCs for upper extremity rigidity were 0.68 (95% CI 0.61–0.75), *P* < 0.001 and 0.61 (95% CI 0.53–0.68), *P* = 0.006 for facial expression (Supplementary Figure).

**Table 2:** Logistic regression analyses of the motor signs and motor symptom duration that differed between patients with and without striatal DAT deficiency in [I-123]FP-CIT SPECT

	Univariate analyses			Multivariate model		
	OR	*P* value	95% CI	OR	*P* value	95% CI
Hypomimia	2.14	0.018	1.14–4.00	2.15	0.025	1.10–4.20
Upper extremity rigidity	4.79	0.006	1.56–14.75	3.34	0.045	1.03–10.86
Motor symptom duration (years)	0.92	0.023	0.86–0.99	0.92	0.026	0.86–0.99

In patients with DAT deficiency, caudate DAT binding was associated with facial expression scores (*F* = 7.51, *P* < 0.001, Fig. 1e). Putamen DAT binding was associated with facial expression (*F* = 4.52, *P* = 0.005) but with only one significant pairwise post hoc comparison (0 vs. 3, *P* = 0.005, 1 vs. 3, *P* = 0.054, 0 vs. 2, *P* = 0.060; the rest comparisons, *P* > 0.32). Upper extremity rigidity was associated with caudate (*F* = 3.88, *P* = 0.011, Fig. 1f) and putamen DAT binding (*F* = 3.17, *P* = 0.027, one significant pairwise post hoc comparison of 1–1.5 vs. 3–4, *P* = 0.024; the rest comparisons, *P* > 0.27). In patients with normal DAT binding, no associations were observed between facial expression and caudate (*F* = 1.21, *P* = 0.31) or putamen DAT binding (*F* = 1.24, *P* = 0.30) or between upper extremity rigidity and caudate (*F* = 1.08, *P* = 0.36) or putamen DAT binding (*F* = 1.49, *P* = 0.22).

**Fig. 2:**
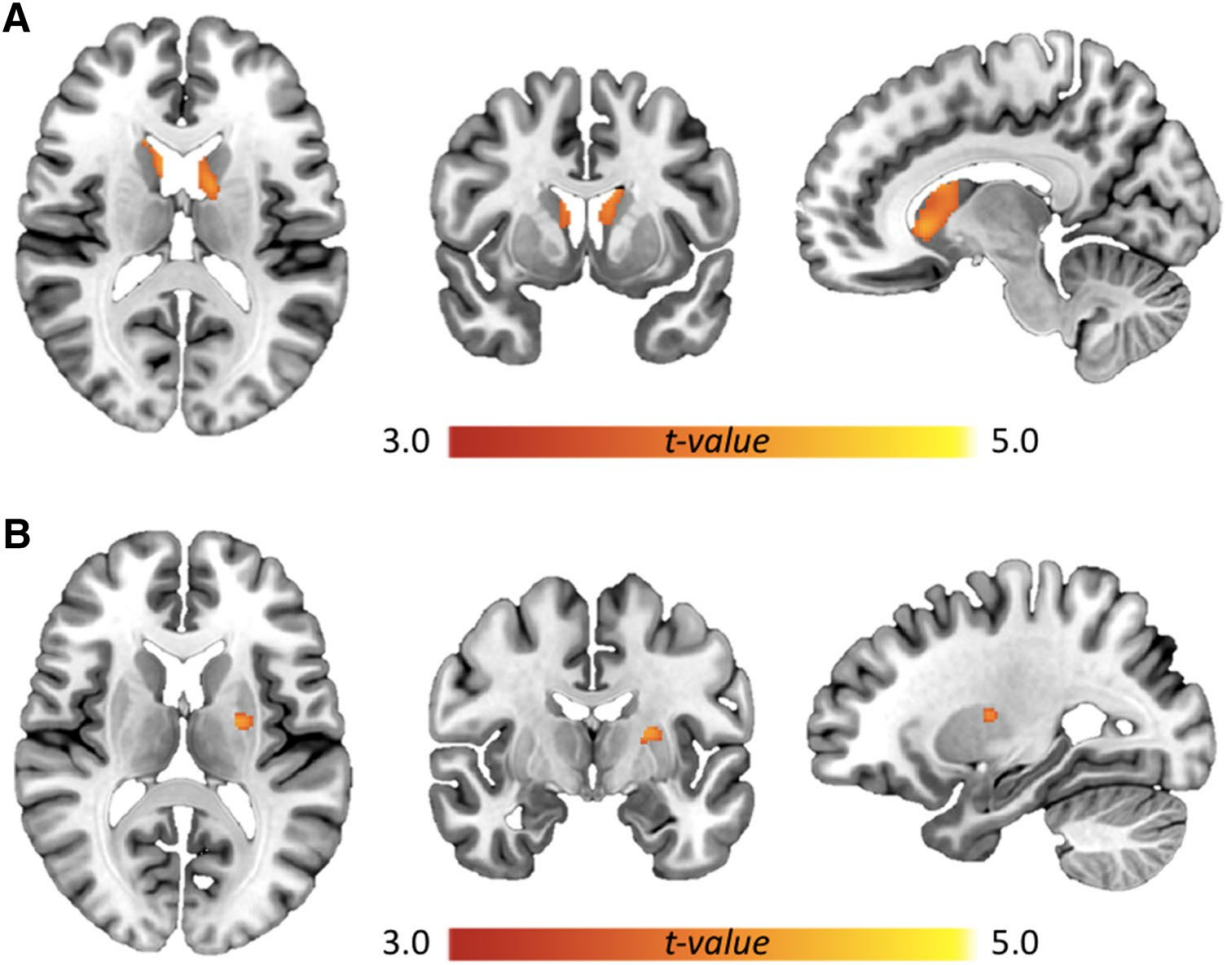
Associations between reduced striatal DAT binding and hypomimia (**a**) and upper extremity rigidity (**b**). Statistical *t* maps including only voxels with voxel-level FWE-corrected *P* < 0.05 are shown and overlaid on the MNI152 T1-weighted template

In the voxel wise analyses, reduced facial expression was associated with reduced DAT binding bilaterally in the caudate nucleus (cluster 1 extent 459 voxels, peak at − 14 − 3 24 with *T*_max_ = 4.51, *P*_FWE_ = 0.003; cluster 2 extent 456 voxels, peak at 9 20 0 with *T*_max_ = 4.31, *P*_FWE_ = 0.006) (Fig. 2a). Upper extremity rigidity was associated with lower DAT binding in the left putamen (cluster extent 74 voxels, peak at − 22 − 8 12 with *T*_max_ = 4.24, *P*_FWE_ = 0.007) (Fig. 2b). When using multivariate regression that included both signs in the model, both the hypomimia (cluster 1 extent 234 voxels, peak at − 14 − 3 24 with *T*_max_ = 4.26, *P*_FWE_ = 0.007; cluster 2 extent 262 voxels, peak at 10 20 0 with *T*_max_ = 3.93, *P*_FWE_ = 0.01) and the upper extremity rigidity (cluster extent 53 voxels, peak at − 26 − 8 − 12 with *T*_max_ = 4.10, *P*_FWE_ = 0.01) effects remained significant.

All of the previous results, except the association between hypomimia and a higher likelihood of DAT deficiency, remained the same in the subsample of patients without antiparkinsonian medications (Supplementary Material). The results remained essentially the same when patients on SSRI/SNRI medications were excluded from the analyses.

## Discussion

The present results indicate that the presence of upper extremity rigidity and reduced facial expression are independently associated with a higher likelihood of striatal dopamine deficiency in patients with parkinsonism or tremor of an unknown origin. On the other hand, overall motor symptom severity or other common parkinsonian motor signs, such as bradykinesia or rest tremor, did not differ between parkinsonism patients with normal and abnormal DAT binding. Furthermore, parkinsonian hypomimia appears to be associated with dopamine degeneration particularly in the caudate nucleus. It should be noted that a longer duration of motor symptoms was associated with a lower likelihood of DAT deficiency. Thus, this result confirms our previous findings, probably reflecting the less progressive nature of parkinsonian symptoms in patients with normal DAT binding [16].

The highest likelihood of DAT deficiency was observed with upper extremity rigidity in at least one body side. The OR was even higher in the subsample of patients who were not receiving antiparkinsonian medications. We are not aware of any previous studies that have investigated the association of upper extremity rigidity and DAT deficiency. The reason why in particular upper extremity rigidity was indicative of DAT loss might be due to many factors that cause and mimic increased lower extremity muscle tone and problems with gait, such as lumbar spine degenerative changes and degenerative arthritis, resulting in apparent lack of differences in the lower extremity rigidity scores between groups. It is of special interest that, unlike most other items on the motor MDS-UPDRS, the estimation of rigidity is not based on inspection but rather requires hands-on examination by the clinician. However, in accordance with earlier results of correlations between DAT and rigidity [7], there were only mild associations between the magnitude of DAT binding and the severity of upper extremity rigidity. This may have been partially due to inter-rater variability in the rigidity severity estimates. Thus, the detection of upper extremity rigidity might serve as a clinical trigger-finding (yes/no) for neurodegenerative parkinsonism disorders associated with dopamine loss even if it cannot be used as a reflector of the magnitude of striatal dopamine deficiency.

A reduction in facial expression, as seen in reduced eye-blink frequency, masked faces, reduced spontaneous smiling and parting of the lips, was also associated with a higher likelihood of DAT deficiency. Hypomimia, the reduction of spontaneous facial movements and emotional facial expressions, is a common feature of neurodegenerative parkinsonism. Previous results have indicated that reduced facial expression in PD could be a reflection of facial bradykinesia that differs from bradykinesia in the limbs, as facial movements are not only voluntary but also involuntary (spontaneous and emotional) [17]. The loss of spontaneous facial expression, such as a reduced blinking rate, has been suggested to be related to central dopamine deficiency and to respond well to dopaminergic medication [18]. On the other hand, the impairment in emotional facial expressiveness seems to correlate with the impairment of facial emotions recognition both in PD [19] and in healthy individuals [20]. It should be noted that, as the association between hypomimia and a higher likelihood of DAT deficiency was not observed in the subsample analyses, there was a possible medication effect in hypomimia. However, the present results, also the subsample analyses, further pointed to a specific association between the decreased facial expression and the reduced dopamine function in the caudate nucleus in patients with abnormal DAT binding. It is of special interest to note that apathy, a common neuropsychiatric symptom in PD, has also been reported to be associated with caudate DAT function in early PD [21]. Given the difficulties in emotional processing of PD patients at multiple levels [22], the impairment in emotional facial expressiveness [19], and the localization of hypomimia in the caudate nucleus and not in the nigrostriatally more relevant putamen, we consider that apathy and hypomimia could represent different but interconnected aspects of abnormal emotional functions in neurodegenerative parkinsonism. Further studies are needed to investigate whether the relationship between facial expression and dopamine is driven more by motor circuitry or by emotional processing.

It is noteworthy that the degree of bradykinesia, the most cardinal feature of parkinsonism and PD [23], did not distinguish parkinsonism patients with normal and abnormal DAT uptake. Thus, the spectrum of neurodegenerative and non-neurodegenerative causes of bradykinesia may clinically appear as similar levels of bradykinesia. Slowness of movements is part of the motor dysfunction in many movement disorders, it can be present in any condition with muscle weakness, and it may also be included in the phenotype of depression [24]. The present results cannot be interpreted to lessen the value of bradykinesia in the diagnostics of PD nor the important role of the slowness of movements in the pathophysiology of parkinsonism. Our finding rather noted the difficulties when one tries to identify patients with dopaminergic degeneration on the basis of bradykinesia. Indeed, the terminology considering bradykinesia and its different dimensions has been highly variable in the earlier literature [25], and it has been speculated that the different dimensions of bradykinesia should be rated separately [23]. A previous blinded video study demonstrated that the clinical symptom-based separation of tremulous patients with and without dopamine deficiency is difficult even for movement disorder specialists, and it was also noted that the true parkinsonian bradykinesia with both slowness and decrement is very hard to diagnose [26]. In line with that study, our study underlines the similarities in tremor signs in a large number of patients by showing that the presence or the magnitude of rest, postural or kinetic tremors has minimal value in predicting striatal dopamine loss in a heterogeneous clinical sample of parkinsonism patients. Not even the tremor asymmetry index was able to differentiate between the two patient groups with and without DAT deficiency in the present study. However, it is noteworthy that the asymmetry of all bilateral motor signs, bradykinesia items and rigidity tended to be more pronounced in patients with abnormal DAT binding, and this finding needs more detailed investigation in the future studies.

The important strengths of the study were the unbiased motor evaluations (not biased by the knowledge of the imaging results) and automated semi-quantitative age- and scanner-corrected ROI analyses, along with voxel-by-voxel analyses. In addition, the scans with semi-quantitative analyses on the borderline of abnormality underwent a separate visual expert evaluation when these scans were categorized into groups of normal and abnormal DAT binding [27]. The distribution of patients with normal and abnormal DAT binding were similar to the earlier retrospective results of patients scanned in Center 1 [28]. Regarding limitations, the study sample consisted of patients who were scanned with SPECT due to clinical diagnostic difficulties, and therefore, the sample was probably not fully representative of typical parkinsonism patients in neurological outpatient clinics. Nevertheless, the sample represents clinical neurological diagnostic reality much better than studies where comparisons were made between patients with established PD diagnoses and healthy controls. The MDS-UPDRS part III scores were somewhat higher when compared to some previous studies of PD patients [9], but this can be explained by the study setting and study sample that included also more advanced parkinsonism patients with also other diagnoses than PD. As a limitation, it should be noted that the MDS-UPDRS rating scale was developed for PD patients in particular and it may not be as suitable for other patient groups. The present results were limited also because they focused on motor function and were derived from motor examinations only, without possibly relevant non-motor predictive signs of striatal DAT loss. Finally, cerebrovascular disease could affect both DAT SPECT imaging and motor symptoms if there are ischemic lesions in relevant regions along the nigrostriatal tract. However, from hospital records we identified only two patients who had striatal DAT deficiency with parallel evidence of possibly relevant vascular lesions.

## Conclusions

The present study demonstrates that the presence of upper extremity rigidity and reduced facial expression, when assessed using the clinimetric properties of the motor MDS-UPDRS, are associated with a higher likelihood of striatal dopamine neurotransmission deficiency in patients with parkinsonism or tremor of an unknown origin. Reduced facial expression appears to be specifically associated with decreased caudate nucleus dopamine function. This could bear relevance not only for motor facial dysfunction, but also for possible pathophysiological emotional mechanisms of hypomimia in neurodegenerative parkinsonism.

## Electronic supplementary material

Below is the link to the electronic supplementary material.

Supplementary material 1 (DOCX 110 KB)

Supplementary material 2 (DOCX 80 KB)

Supplementary material 3 (DOCX 92 KB)

Supplementary material 4 (TIFF 922 KB)
