# Supplementary material for: Individual parkinsonian motor signs and striatal dopamine transporter deficiency: a study with [I-123]FP-CIT SPECT
Source: J Neurol. 2019 Jan 28;266(4):826–34. doi: 10.1007/s00415-019-09202-6 (PMC6420881; doi:10.1007/s00415-019-09202-6)
Supplement: Supplementary file 1 — Supplementary material 1 (DOCX 110 KB) [file 415_2019_9202_MOESM1_ESM.docx]

### **Supplementary Material**

### **METHODS**

### **Patients and study description**

### The reasons for scanning were to aid the clinical diagnostics by the treating physicians, most often general neurologists, due to suspected PD (n=167) or atypical parkinsonism (n=27), or the differential diagnosis between PD and ET (n=8), PD and DIP (drug-induced parkinsonism) (n=9), PD and VP (n=3), DLB and Alzheimer’s disease (AD) or other dementias (n=6), and secondary parkinsonism due to brain tumor or PD (n=1). All clinical investigators involved in the clinical ratings had passed the MDS-UPDRS-III Training Program and Exercise [1]. For ten patients with randomly missing items on the MDS-UPDRS-III (≤5 items per patient), the MDS-UPDRS-III total scores were adjusted according to the number of missing items, as described previously [2].

### **SPECT Imaging**

To prevent radiation exposure of the thyroid tissue, 250-300 mg of potassium perchlorate was given 30-60 min before the tracer injection. The injected activity of [I-123]FP-CIT was 185 MBq, and the radiopharmaceutical was administered using a slow 20-s intravenous injection. The image acquisition started 3 h after the injection. In Centre 1, all patients were scanned using two GE Infinia II Hawkeye SPECT/CT scanners (GE Healthcare, Tirat Hacarmel, Israel). In Centre 2, 41 patients were scanned using Philips Brightview XCT (Philips Healthcare, Eindhoven, The Netherlands), and two patients were scanned using a Siemens Symbia T2 (Siemens Healthineers, Erlangen, Germany) SPECT/CT scanner. All SPECT/CTs were dual-head scanners with low-energy high-resolution collimators. Patients were positioned to lie supine, and the scanner heads were adjusted close to each patient’s head in a circular orbit. For the GE Infinia and the Brightview, the energy window was 159 keV ± 10 % and for the Symbia, the energy window was 159 keV ± 7.5 %. For all scanners, the acquisition matrix size was 128x128, the rotation arc per each scanner head was 180° in step-and-shoot mode, and the angular step was 3°, resulting in 60 projections for each scanner head and a total of 120 projections. For the Brightview and Symbia, the acquisition zoom was 1.46, and the time per projection was 30 s. The corresponding values for the GE Infinia were 1.25 and 30 s.

**SPECT Image Analyses**

SPECT images were reconstructed using a three-dimensional (3D) ordered-subsets expectation maximization (OSEM) reconstruction algorithm (HybridRecon Neurology, version 1.3, Hermes Medical Solutions AB, Stockholm, Sweden), with 16 iterations, 4 subsets, uniform attenuation correction with the attenuation coefficient of 0.146 1/cm, collimator response correction using Gaussian diffusion model, Monte-Carlo-based scatter correction for the I-123 isotope and 3D Gaussian postfiltering with a full-width-at-half-maximum of 0.7 cm. The reconstructed images were analyzed using the BRASS analysis software.

Before the first patient was scanned, all SPECT scanners were similarly calibrated using a striatal phantom to minimize the effect of sensitivity variations between the scanners on the acquired data [3]. The camera-specific calibration coefficients were then implemented into BRASS software. With BRASS software, the SBRs of DAT binding were calculated over six striatal ROIs (the right and left anterior putamen, the right and left posterior putamen, the right and left caudate nucleus), which were automatically segmented. The occipital cortex was used as the reference region and the SBRs were calculated as: SBR = (ROI – ROI_occipital_) / ROI_occipital_ [4]. The mean caudate DAT binding and mean putamen DAT binding (Supplementary Table 1) were selected as the primary SPECT variables in the statistical analyses of the ROI data.

The spatial distribution of the clinical correlates of the [I-123]FP-CIT SBR within the striatum was investigated with a voxel-based approach using SPM 12. First, the images fitted to the standard template implemented in BRASS were averaged, and the average SBR image was used to calculate nonlinear registration to the in-house [I-123]FP-CIT template in the MNI152 standard space [5]. The registration parameters were then used to warp all individual SBR images to the MNI152 space for voxelwise statistical analyses. The analyses were restricted to the striatum by using a previously published striatal mask [6].

**RESULTS**

There were no significant differences in the mean worse side posterior putamen DAT binding between different scanners (P=0.37) or between patients who were and were not receiving anti-parkinsonian medications [non-significant in the abnormal (P=0.23) and normal (P=0.42) striatal DAT binding groups].

**Separate analyses of patients not receiving antiparkinsonian medications**

Out of these 181 patients, 77 showed abnormal DAT binding and 104 had normal DAT binding. The patients with abnormal and normal DAT binding differed in motor symptom duration (P<0.001), rigidity total score (P=0.006), upper extremity rigidity total score (P<0.001), and facial expression (P=0.041), but the difference in facial expression did not survive the Benjamini-Hochberg procedure. Unilateral kinetic tremor of upper extremity (P=0.039), asymmetry index of all lateralized motor signs (P=0.022) and rigidity asymmetry index (P=0.029) also suggested differences between the groups, but they did not survive the Benjamini-Hochberg procedure.

The presence of any upper extremity rigidity was associated with a higher likelihood of striatal DAT deficiency (OR 7.33, 95% CI 1.69-32.75, P=0.009). Hypomimia was not anymore significantly associated with a higher likelihood of striatal DAT deficiency (P=0.21). Each one-year increase in the motor symptom duration was associated with a lower likelihood of DAT deficiency (OR=0.77, 95%CI 0.66-0.91, P=0.002).

In patients with DAT deficiency, caudate DAT binding was associated with facial expression scores (F=7.13, P<0.001, pairwise comparisons 0 vs. 1, P=0.018; 0 vs. 2. P=0.002; 0 vs. 3, P=0.001; 1 vs. 2, P=0.92; 1 vs. 3, P=0.18; 2 vs. 3, P=0.33). Putamen DAT binding was associated with facial expression scores but again with only one significant pairwise comparison (F=3.29, P=0.025, pairwise comparisons 0 vs. 3, P=0.033, the rest comparisons, P>0.09). Upper extremity rigidity was associated with caudate DAT binding with one significant pairwise comparison (F=3.78, P=0,14, pairwise comparisons 1 vs. 3, P=0.027, the rest comparisons P>0.12) and with putamen DAT binding with one significant pairwise comparison (F=3.66, P=0.16, pairwise comparisons 1vs.3, P=0.030, the rest comparisons P>0.11). In patients with normal DAT binding, no associations were observed between facial expression and caudate (F=0.56, P=0.64) or putamen (F=0.97, P=0.40) DAT binding or between upper extremity rigidity and caudate (F=0.55, P=0.65) or putamen (F=0.98, P=0.40) DAT binding.

In the voxelwise analyses, all results remained significant when excluding patient with current antiparkinsonian medication.

## **REFERENCES**

[1] Goetz, C.G., Stebbins, G.T., Chmura, T.A., Fahn, S., Poewe, W. and Tanner, C.M. Teaching program for the Movement Disorder Society-sponsored revision of the Unified Parkinson's Disease Rating Scale: (MDS-UPDRS). *Mov Disord* 2010; **25**:1190-1194.

[2] Goetz, C.G., Luo, S., Wang, L., Tilley, B.C., LaPelle, N.R. and Stebbins, G.T. Handling missing values in the MDS-UPDRS. *Mov Disord* 2015; **30**:1632-1638.

[3] Tossici-Bolt, L., Dickson, J.C., Sera, T., de Nijs, R., Bagnara, M.C., Jonsson, C., Scheepers, E., Zito, F., Seese, A., Koulibaly, P.M., Kapucu, O.L., Koole, M., Raith, M., George, J., Lonsdale, M.N., Münzing, W., Tatsch, K. and Varrone, A. Calibration of gamma camera systems for a multicentre European ¹²³I-FP-CIT SPECT normal database. *Eur J Nucl Med Mol Imaging* 2011; **38**:1529-1540.

[4] Joutsa, J., Johansson, J. and Kaasinen, V. Is Occipital Cortex a Valid Reference Region in 123I-FP-CIT SPECT Imaging? *Clin Nucl Med* 2015; **40**:615-616.

[5] Kaasinen, V., Joutsa, J., Noponen, T., Johansson, J. and Seppänen, M. Effects of aging and gender on striatal and extrastriatal [(123)I]FP-CIT binding in Parkinson's disease. *Neurobiol Aging* 2015; **36**:1757-1763.

[6] Choi, E.Y., Yeo, B.T. and Buckner, R.L. The organization of the human striatum estimated by intrinsic functional connectivity. *J Neurophysiol* 2012; **108**:2242-2263.
