# Supplementary material for: Individual parkinsonian motor signs and striatal dopamine transporter deficiency: a study with [I-123]FP-CIT SPECT
Source: J Neurol. 2019 Jan 28;266(4):826–34. doi: 10.1007/s00415-019-09202-6 (PMC6420881; doi:10.1007/s00415-019-09202-6)
Supplement: Supplementary file 2 — Supplementary material 2 (DOCX 80 KB) [file 415_2019_9202_MOESM2_ESM.docx]

**Supplementary Table 1** Explanations of the motor MDS-UPDRS and [I-123]FP-CIT SPECT variables.

| Bradykinesia total score | Finger tapping, hand movements, pronation-supination, toe tapping, leg agility and global bradykinesia in MDS-UPDRS-III |
| --- | --- |
| Rigidity total score | Rigidity in neck, right and left upper extremities, right and left lower extremity in MDS-UPDRS-III |
| Tremor total score | Postural, kinetic and rest tremor of upper extremities, rest tremor of lower extremities, lip/jaw rest tremor and constancy of rest tremor in MDS-UPDRS-III |
| Axial signs | Arising from chair, gait, freezing of gait, postural instability and posture in MDS-UPDRS-III |
| Asymmetry index | (Score of lateralized motor items in MDS-UPDRS-III right side – Score of lateralized motor items left side) / (Right side + Left side) (absolute value) |
| Asymmetry index (bradykinesia) | (Score of bradykinesia items right side – Score of lateralized bradykinesia items left side) / (Right side + Left side) [absolute value] |
| Asymmetry index (rigidity) | (Score of rigidity right side – score of rigidity left side) / (right side + left side) (absolute value) |
| Asymmetry index (tremor) | (Score of lateralized tremor items right side – Score of lateralized tremor items left side) / (Right side + Left side) (absolute value) |
| Putamen DAT binding | {[(Posterior putamen SBR right + anterior putamen SBR right) / 2] + [(Posterior putamen SBR left + anterior putamen SBR left) / 2]} /2 |
| Caudate DAT binding | (Caudate SBR right + caudate SBR left) / 2 |
| Specific binding ratio | SBR = (ROI – ROI_occipital_) / ROI_occipital_ |

DAT = dopamine transporter
ROI = region of interest
