# Supplementary material for: Individual parkinsonian motor signs and striatal dopamine transporter deficiency: a study with [I-123]FP-CIT SPECT
Source: J Neurol. 2019 Jan 28;266(4):826–34. doi: 10.1007/s00415-019-09202-6 (PMC6420881; doi:10.1007/s00415-019-09202-6)
Supplement: Supplementary file 3 — Supplementary material 3 (DOCX 92 KB) [file 415_2019_9202_MOESM3_ESM.docx]

**Supplementary Table 2** Presence of tremor and motor symptom asymmetry in 221 patients with parkinsonism of an unknown origin with and without striatal dopamine transporter (DAT) binding deficiency in [I-123]FP-CIT SPECT.

|  | ***DAT Normal***  ***n = 111*** | ***DAT Abnormal***  ***n = 110*** | ***P* *value*** |
| --- | --- | --- | --- |
| ***Tremor*** |  |  |  |
| Unilateral kinetic tremor of upper extremity | 20 (18.0) | 32 (29.4)^1^ | 0.048 |
| Kinetic tremor of both upper extremities | 64 (57.7) | 48 (44.0)^1^ | 0.043 |
| Unilateral postural tremor of upper extremity | 27 (24.3) | 35 (31.8) | 0.22 |
| Postural tremor of both upper extremities | 57 (51.4) | 49 (44.5) | 0.31 |
| Unilateral rest tremor of upper extremity | 28 (25.2) | 38 (34.9)^1^ | 0.12 |
| Rest tremor of both upper extremities | 34 (30.6) | 21 (19.3)^1^ | 0.052 |
| Unilateral rest tremor of lower extremity | 8 (7.2) | 13 (11.9)^1^ | 0.23 |
| Rest tremor of both lower extremities | 6 (5.4) | 8 (7.3)^1^ | 0.56 |
| Lip and Jaw Rest tremor | 15 (13.5) | 13 (11.8) | 0.71 |
| ***Asymmetry of motor symptoms***^2^ |  |  |  |
| Asymmetry index (total) | 0.19 (0.20) | 0.27 (0.26) | 0.015 |
| Asymmetry index (bradykinesia) | 0.21 (0.25) | 0.30 (0.30) | 0.027 |
| Asymmetry index (tremor) | 0.31 (0.35) | 0.41 (0.41) | 0.20 |
| Asymmetry index (rigidity) | 0.19 (0.30) | 0.25 (0.30) | 0.014 |
| Asymmetry index (rigidity upper extremities) | 0.24 (0.39) | 0.26 (0.33) | 0.033 |

Mann Whitney U-tests and Chi-Square tests were used to investigate differences between groups. Values are n (%) or mean (SD) for demonstrative purposes. None of the comparisons were significant after multiple comparisons correction using Benjamini-Hochberg procedure.

^1^One missing value.

^2^Mann-Whitey U-test.
