# Supplementary figures and images for: Individual parkinsonian motor signs and striatal dopamine transporter deficiency: a study with [I-123]FP-CIT SPECT
Source: J Neurol. 2019 Jan 28;266(4):826–34. doi: 10.1007/s00415-019-09202-6 (PMC6420881; doi:10.1007/s00415-019-09202-6)

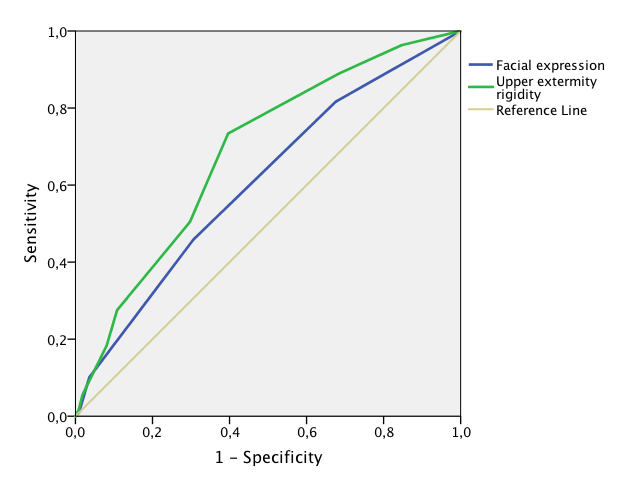

Supplement: Supplementary file 4 — Supplementary material 4 (TIFF 922 KB) [file 415_2019_9202_MOESM4_ESM.tiff]
